# Debates on family planning and the contraceptive pill in the Irish magazine Woman’s Way, 1963–1973

**DOI:** 10.1080/09612025.2020.1833495

**Published:** 2020-10-29

**Authors:** Laura Kelly

## Abstract

This article explores discussions of family planning and the contraceptive pill in the popular Irish women’s magazine *Woman’s Way* between 1963 and 1973. Contraception was criminalised in Ireland in 1935 and literature relating to birth control was banned under the 1929 Censorship of Publications Act. The contraceptive pill was marketed as a cycle regulator from 1963 until legalization in 1979. This article outlines how women accessed the contraceptive pill, the geographical and class inequalities around this access and how *Woman’s Way* was an important vehicle for debates around the pill. The article assesses what discussions of the contraceptive pill can tell us about marriage dynamics, the role of sympathetic doctors, the power of the Catholic Church. Ultimately, it illustrates how the magazine was an important source of information on family planning for Irish women and how it also enabled women to air their views on the issue.

In 1973, Irish magazine *Woman’s Way*, published an article discussing the experiences of women living in fear of unplanned pregnancy from month to month. Joan, a twenty-seven-year-old woman, was described as living in ‘a state of constant fear of another pregnancy’. She explained to the journalist:

It’s one long worry. Another child would be disastrous because we just cannot afford it what with the high cost of living, not to mention being too busy to even think. Because of the fear of another pregnancy, we don’t even sleep together. Consequently we are not living a normal married life, a natural life. It’s as simple as that.^1^

Joan’s testimony was not atypical, and like many accounts published in the magazine in the 1960s and 1970s, provides an insight into the lived reality for many Irish women without legal access to contraception. For these women, lack of access to contraception meant considerable anxiety each month around falling pregnant, and placed a significant strain on marital relationships. Contraception was made illegal by the Irish government in 1935 with the introduction of the Criminal Law Amendment Act that forbade the import, sale, and advertisement of contraceptives, and it would not be legalized until 1979.^2^

Irish men and women also struggled to obtain basic information on contraception in the form of books or leaflets as the 1929 Censorship of Publications Act banned literature on sex and contraception and books deemed to advocate contraception or abortion.^3^ This included a 1976 publication by the Irish Family Planning Association with basic information on sex and family planning, which the Act deemed ‘obscene’. Thus, Irish birth rates remained high. For instance, in 1961, the figure for legitimate births per married women in Ireland was 195.5 per thousand, almost double the figure in England and Wales (108.3).^4^ Despite the number of births declining by 3 percent in the 1960s, marital fertility in Ireland remained out of line with other Western countries where fertility rates were falling sharply.^5^ Thus, in 1967, 23 percent of women giving birth in the National Maternity Hospital in Dublin gave birth to their fifth child.^6^ In Ireland the total fertility rate (live births per woman) was 3.85 in 1970, compared to 2.38 in Italy, and 2.25 in Belgium.^7^ Ultimately, however, over the twentieth century, Ireland experienced long-term population decline, high rates of emigration as well as low rates of marriage and late age of marriage, high marital fertility and a late transition to smaller families.^8^

Despite the illegality of contraception in Ireland, some women did have access to the contraceptive pill, first prescribed in the country in 1963. Because pharmaceutical companies initially marketed the pill as a cycle regulator, users of the pill were able to navigate the Irish ban on contraception by requesting it for menstrual irregularities.^9^ At a press conference held by Syntex Pharmaceuticals in Cork in June 1967, Dr. Anthony Jarrett, medical director of the firm, estimated that about 3 percent of Irish women between the age of 16 and 45 were using the contraceptive pill, in contrast with approximately 10 percent of women in the United Kingdom.^10^ The following year, the marketing director of Syntex, Ronald Levin, explained that the pill was ‘used for therapeutic as well as social reasons, and it is impossible to distinguish between packs sold for different reasons’. However, Levin believed, from ‘the conversations we’ve had with doctors in the Republic … that the majority of general practitioners in Ireland are prescribing the Pill for social reasons’.^11^ According to one Dublin gynecologist in March 1968,

more and more now are going on to the Pill … certainly, the Pill is making the big breakthrough in Ireland now. More and more general practitioners are prescribing it, and very few doctors would refuse it now to any woman who asks for it.^12^

This suggests that by the late 1960s, attitudes among doctors with regard to the contraceptive pill were starting to shift. Yet, as I will argue, women’s access to the contraceptive pill relied on the support of a sympathetic doctor who would prescribe it.

Recent scholarship has highlighted the role of the Catholic Church hierarchy, Irish government, and the medical profession in debates surrounding contraception in Ireland in the twentieth century. Lindsey Earner-Byrne, for instance, has shown how the medical profession in the early twentieth century ‘used papal teaching to bolster their arguments against any form of state medicine and to promote Catholic control of medicine’.^13^ But this historiographical focus on the role of the medical profession and institutional reform has neglected or marginalized the experiences of ‘ordinary’ women and of grassroots activists.^14^ The recent repeal of the eighth amendment in Ireland in May 2018, which legalized abortion, demonstrates the necessity of understanding the longer history of reproductive rights in Ireland, and in particular, the period of the contraception ban.^15^ Abortion was illegal in Ireland under the 1861 Offences against the Person Act and later under the 1983 eighth amendment, although it was possible for Irish women to travel to Britain after the decriminalization of abortion there in 1967.^16^ Recent important studies by Cara Delay, Leanne McCormick, Lindsey Earner-Byrne and Diane Urqhuart have highlighted the impact of legal restrictions on the experiences of ordinary women wishing to access abortion in twentieth-century Ireland.^17^ In order to further redress this balance, this article will examine the voices and experiences of ‘ordinary’ women in relation to contraception access through a focus on the popular Irish women’s magazine *Woman’s Way*.

*Woman’s Way* was a weekly magazine that focused on women’s lives.^18^ When Caroline Mitchell, a Protestant, became editor in 1965, the editorial line was broadly in favor of birth control.^19^ The magazine was aimed at women of all ages and was competitively priced at between 8d and 10d during the 1960s, which meant that it was affordable for working-class women.^20^ As Caitriona Clear’s valuable work in this area has shown, women’s magazines are an important tool for exploring the experiences of Irish women in the twentieth century.^21^ A significant feature of the magazine was its problem pages, or advice columns, where women would write in seeking advice on personal matters, and its letter columns, where women could express their opinions on a variety of topics. As Ciara Meehan has shown, Irish women’s magazines had an important role in educating their readers on issues relating to sex and the body.^22^

As Clear notes, it is impossible to determine reliable circulation figures for Irish magazines prior to the 1980s.^23^ However, it is evident that the letters pages were popular. From April 1963 to December 1969, for example, she has calculated that readers sent approximately 1186 problems to *Woman’s Way*, and around 1826 letters on other topics.^24^ While the historian should exercise care when utilizing these sources, as Clear notes, ‘they can, however, be taken with all due caution as representative of the problems that some people felt they could articulate and write about to a magazine and that a magazine could publish’.^25^ More recently, Tracey Loughran has shown how mass-market women’s magazines can act as ‘an invaluable resource for understanding women’s lives in the postwar period’.^26^ Her work has shown how women’s magazines allow the historian to uncover women’s experiences of infertility, particularly within the problem pages of these magazines.^27^ Similarly, in Spain, as Teresa Ortiz-Gomez and Agata Ignaciuk have shown, ‘the print media played a significant role in the popularization of the contraceptive pill during the period when it was legally banned’ and also helped to contribute to ‘a lively social debate about contraception that challenged the reproductive politics of the authoritarian regime’.^28^ In the Spanish context, the openness and plurality of discussions around family planning ‘marked out a “splendid time” of growing plurality within the Catholic community’ and reflected ‘a growing distance between the hierarchy, parts of the clergy and secular Catholics’.^29^ These debates ‘had the effect of moralizing, sanitizing, medicalizing and eventually legitimizing wider social interest in birth control’.^30^

Indeed, as I will show, it is evident that there was a similarly lively debate in the Irish press in the period on the topic, and *Woman’s Way* enabled women to voice their experiences living under the ban on contraception. But the magazine also provided a valuable source of information on family planning in the period when access to family planning advice was restricted. Because the pill was removed from the act of sex, unlike other contraceptives such as condoms (which also had an association with venereal disease), it was easier for it to be discussed in the magazine than these types of contraceptives. In particular, women’s discussions of the pill in Ireland indicate the power of both the medical profession and the Catholic Church over women’s reproductive choices, but also shed light on the importance of issues such as class, location, and marriage dynamics.

This article first discusses how women accessed the contraceptive pill in Ireland, and the class and geographical inequalities that dictated this access. It then outlines how *Woman’s Way* was an important vehicle for debates around the pill. I also assess what discussions of the contraceptive pill can tell us about marriage dynamics, the role of sympathetic doctors, and the power of the Catholic Church. The final part of the article explores the impact of the Catholic encyclical *Humanae Vitae* on women’s opinions of the contraceptive pill, and how the introduction of the encyclical led to an intensification of debates around the issue of conscience.

## Accessing the contraceptive pill

Although the contraceptive pill became available in Ireland from 1963, access remained difficult for the majority of Irish men and women. From that year, the National Maternity Hospital in Dublin established a family planning service to provide advice ‘in conformity with Catholic moral teaching’, and by 1965, all three of the Dublin maternity hospitals were operating ‘Marriage Guidance Clinics’.^31^ Two of the three major maternity hospitals in Dublin, the Coombe Lying-in Hospital and the National Maternity hospitals were Catholic hospitals, with Archbishop John Charles McQuaid acting as governor of their boards.^32^ These clinics provided advice on the rhythm method of family planning, whereby couples were encouraged to avoid sexual intercourse during days of the month when the woman was believed to be fertile but also until regular menstruation returned after the birth of a child.

In an article published in the *Journal of the Irish Medical Association* in 1967, the two doctors in charge of the clinic at the National Maternity Hospital, Dr. Declan Meagher and Dr. Dermot MacDonald, highlighted the problems with the existing law in Ireland. A survey of 107 clinic patients on their previous family planning methods found that 40 percent had previously used *coitus interruptus*, 24 percent calendar methods, 33 percent none, and 3 percent complete abstinence.^33^ The two doctors explained the issues with the calendar method (or safe period), primarily that it required a couple to abstain from intercourse until regular menstruation had returned, which could be five to six months after birth. And ‘since couples are advised to refrain from intercourse in the last two months of pregnancy the use of the rhythm method imposes an intolerable strain on many marriages’. They explained that the policy of the clinic was to prescribe ‘the pill’ as a contraceptive for ‘selected medical and social cases’ and emphasized that advice on family planning should be freely available to all mothers with fear of pregnancy being detrimental to marital harmony.^34^

In 1968, the *Irish Times* estimated that there was a 50 percent increase in the usage of the pill in Ireland, with four anovulant brands available in 1966 and at least ten in 1967. Pharmaceutical representatives who were interviewed for the newspaper suggested that the majority of women were using the pill for ‘social reasons’ and that doctors were anxious for clarification on the Church’s stance on the issue.^35^ Following the 1968 publication of *Humanae Vitae*, the papal encyclical that declared that all contraception, with the exception of natural family planning methods, went against Church teaching, the National Maternity Hospital no longer prescribed the pill. In a July 1968 letter written by Dr. Kieran O’Driscoll, master of the hospital, he assured the archbishop that the ‘Papal Encyclical is accepted as an explicitly clear directive on the subject of birth control, which will be adhered to’.^36^

While it was not possible for women to obtain the pill through the National Maternity Hospital family planning service after 1968, some women could have it prescribed to them through a sympathetic general practitioner (GP). Dublin-based, middle-class women could obtain it through the Fertility Guidance Company clinic. This was a voluntary family planning clinic established in 1969, which provided both medical and non-medical contraceptives (such as condoms and diaphragms) to patients who paid a ‘donation’ rather than a fee. However, the clinic predominantly catered to the urban middle classes, and largely speaking, across Ireland, working-class and rural-based men and women struggled to get access to all forms of contraception. In addition, access depended on a pharmacist who was willing to dispense contraception.

Evidently, access to contraception depended heavily on one’s class and geographic location and *Woman’s Way* publicized these inequalities. A 1968 article by Monica McEnroy, a Catholic mother, nurse, and midwife, deplored ‘the scandal of effective conception control being available, at once and without question, to wealthy city women while the rest of those who want to avail of the mercies of modern medicine are treated as if they were looking for a loan of the consultant’s inside shirt – a mixture of distaste and indignation’.^37^ Mrs. Kearney, the mother of three children, who had been refused the contraceptive pill by her doctor, wanted ‘to have the same facilities for living her married life in peace and harmony with her husband and three children as her sister in England’. Kearney stated that ‘no hospital has the right to make me obey these regulations. I am the one to decide what is necessary for my family’.^38^ Similarly, in the same year, McEnroy reported on the class and geographic inequalities in relation to access to contraception, with women who are ‘rather poor’ or who ‘live in a country area’ suffering from lack of access.^39^ Ultimately, as McEnroy explained, with regard to the existing inequalities facing Irish women in rural areas:

It is wrong and cruel that Dublin women should be able to live happily married lives while women in other parts of Ireland get no chance at all of holding their family welfare together. There is no question whatever of this being unethical. We are constantly receiving appeals for help. We are satisfied that some women are not getting fair play. We cannot know what the situation is exactly, but it is clear they want no more leaflets or books on the safe period. If they lived on the Ulster side of the border [Northern Ireland] they could walk into a family planning clinic. Any woman who wants the pill and is medically suitable has a right to look for it. She also has a right to other effective means of conception control if she prefers.^40^

McEnroy also backed up her arguments with interviews with Irish women in rural areas, including one with thirty-six-year-old Angela, who McEnroy described as living on a farm. Angela had had six children in eight years and first obtained the pill while visiting her sister in Devon, England. Angela told McEnroy ‘It is a nuisance having to go Dublin every six months to get a check-up but sure it is only twice a year after all. I would not say a word about it around here’.^41^ Her quote indicates that it was possible for some rural-based women to gain access to the pill by traveling to Dublin but also sheds light on the potential stigma they faced in doing so, and the fact that she felt that she could not discuss it within her rural community.

Access to contraception continued to be differentiated by class well into the 1970s. In a series of letters published in the *Irish Times* in 1970 on the issue of contraception, readers gave their views. One reader explained that

while the State laws regarding contraception are undoubtedly a denial of a basic human right for those of us who are not Catholics, they do not seriously interfere with the better-off and better-informed, who find it easy enough, if inconvenient to get round them. It is the less well-off who are penalized, who cannot visit England or Belfast, or even perhaps Dublin, where proper contraceptive advice is available, if you know where to find it’.^42^

Indeed, the Church of Ireland had suggested that birth control might be permissible in certain circumstances following the Lambeth Conference in 1930.^43^ Inequalities persisted into the 1970s. As Betty Hilliard has shown, the Catholic Church held significant control over women’s reproductive experiences in the 1950s, 1960 and 1970s. Her interviews with 101 Irish married mothers in the mid-1970s in a settled area of social (public) housing in Cork City illustrates that ‘talk of sexual relations tended to be inextricably linked to an awareness of the likely consequences’.^44^ Only one of the mothers Hilliard interviewed had gained access to the contraceptive pill from her Protestant doctor, while one other mother was introduced to the coil by another Protestant doctor after having eight children. Nonetheless, her husband refused to sign the consent form. However, for the majority of mothers interviewed, there was no way of gaining access to artificial contraception.^45^

## Debates surrounding the contraceptive pill: doctors, religion, and marriage dynamics

In Ireland, as in other Catholic countries with similar legal and religious restrictions on birth control, the contraceptive pill featured heavily in media debates surrounding family planning. In Spain, for example, the first references to the contraceptive pill appeared in publications from 1964.^46^ In the Spanish press, physicians dominated the debate on the contraceptive pill in the 1960s and 1970s, and their focus tended to be on its side effects.^47^ In the publication *Triunfo*, the focus was on the positive aspects of the pill and its role in ‘responsible parenthood’.^48^ In Poland, where there was no ban on contraception, information about the pill had circulated in popular magazines such as *Przyjaciolka* since 1960.^49^ In Portugal during the same time period, debates over the contraceptive pill highlighted the dissatisfaction of a significant proportion of Portuguese elites towards Catholic authority over matters relating to sexuality.^50^

Magazines such as *Woman’s Way* played an important role in illuminating the experiences of 'ordinary' Irish women and their views on the ban on contraception, as well as the importance of marriage dynamics, sympathetic doctors, and the Catholic Church. Discussion of the contraceptive pill first appeared in *Woman’s Way* in 1963 and articles and letters highlight a range of perspectives on the topic. Angela Macnamara, a columnist who answered letters to the magazine’s advice column, represents more conservative or Catholic views on contraception. Paul Ryan’s engaging study of Macnamara’s agony aunt column in the *Evening Press* from 1963 to 1980 shows how her column ‘was one of the few sources of sexual information in Ireland especially during the 1960s’.^51^ In a 1963 article on artificial forms of contraception, Angela Macnamara explained that ‘contraceptive practices kill love and are an insult to the Creator’. ^52^ Macnamara’s stance was firmly rooted in Catholic teachings, but she was also concerned about outside influences that would tempt vulnerable young Irish women. She suggested that parents should ensure that their children

going abroad must know the evils that exist, the reasons for and the methods of avoiding them […] many an Irish girl abroad has been introduced to the evils of contraception under the guise of ‘the modern way of life’. Her innocence and ignorance are mocked and exploited. Without realizing it, she falls victim to a way of life leading insidiously away from God and from everything she valued at home.^53^

It seems here that Macnamara is alluding to the potential for young Irish women to be influenced by ideas around contraception in the United Kingdom, where contraceptives and advice on family planning could be legally obtained. This was a long-standing concern, and the fate of single women who migrated to Britain was the attention of public and moral discourse throughout the twentieth century.^54^ In addition, during the 1960s, sexual themes and issues such as contraception and divorce became an increasingly visible part of popular culture as a result of imported television programs and popular music.^55^ Moreover, the fact that Macnamara emphasized Irish youth is important: there was a fear among those opposed to contraception that access to it would result in increased promiscuity in young people. Macnamara opposed the use of artificial forms of contraception by both married and single men and women.^56^ However, her 1963 piece was not typical of subsequent articles on the topic, which tended to focus on the experiences of women who could not access contraception and the benefits of the pill. Yet, in responses to letters to her problem page, she remained consistent in her views, and suggested that individuals wishing to space their children should abstain from sexual intercourse during fertile periods. For instance, one ‘Teenage Girl’ writing in 1965 to Macnamara asked, ‘Isn’t it unfair that Catholics are expected to have as many children as possible? Why is this so?’ Macnamara responded:

It is *not* so. The duty of parents is not only to bring children into the world, but also to provide for them, educate them, give them the best possible chance in life and of getting to heaven. For reasons of health, finance and social conditions parents are often unable to do all they would like for their children unless they regulate the number they have. This can be done by abstaining from sexual intercourse and parents require specialized advice from a doctor regarding the times to abstain, as the fertile period varies from one individual to another.^57^

Macnamara acknowledged the financial problems facing some parents raising large families, but she only advocated the safe period as a form of family planning, although often with caution.^58^

For Ryan, ‘Macnamara’s power lay in the monopoly of information about sex that she held over a section of the population, particularly in 1960s Ireland’.^59^ In response to letters asking her for advice on contraception, she never recommended artificial forms of contraception. For example, a reader writing to Macnamara in 1967 asked: ‘What is meant by contraceptives? Can a woman take anything to stop ovulation if the couple do not want to have another child for some time?’ Macnamara responded that ‘the pill […] is, according to the present teaching of the Catholic church, against the law of nature and of God’, and stated that if a married couple wished to space their family, they should do so by using the infertile period.^60^

In 1965, a full article devoted to the topic of ‘regulation of family’ appeared in *Woman’s Way* by the magazine’s marriage counselor, who was described as a woman doctor. The article began by stating that the counselor had received ‘so many letters’ asking for advice about family planning that it necessitated a full article on the topic. She explained:

It seems such a shame that when a woman says she is about to have another child, instead of the event being heralded with joy, it is met with sympathy. It is not surprising that so many women regard the possibility of becoming pregnant with a real anxiety which is often made worse by the uncertainties about the real teachings of the Catholic Church on this subject.^61^

While the article did not advise the use of the pill, it did discuss the benefits of the rhythm method, and how this could be used to ‘lead to a holy and happy marriage’.^62^

As well as discussing the benefits of natural methods of family planning in its articles, *Woman’s Way* also carried advertisements for devices to aid it. In 1965, the magazine ran an advertisement for the CD (Conception Days) indicator, a device used to facilitate the rhythm method. The device had a numerical display that allowed the woman to rotate the numbers to the date when menstruation began to pinpoint her fertile days. This advertisement incited the anger of the archbishop of Dublin John Charles McQuaid, who responded ‘I never approve of any device and do not approve of this’.^63^ Despite this backlash, an advertisement for the CD indicator again appeared in a September 1966 issue. The announcement described it as a ‘family planning device TESTED AND APPROVED by Professor Ogino and Professor Knaus, the eminent gynecologists, who developed the Rhythm Method’. An image of the device showed a woman’s hand with a wedding ring holding it with a form for readers to send for a brochure giving details of the device. The form was captioned ‘It costs a little more – but you are much safer’.^64^ In October of the same year, an advertisement for the ‘Ovula’ also appeared, a thermometer ‘specially designed with an easy to read scale’, which ‘will clearly indicate the date of Ovulation and the limits of the Safe Period’.^65^ In November 1966, the magazine also advertised a device called the ‘Menorota’, which claimed it allowed users to ‘CONFIDENTLY learn the safe and fertile days in any “month” – even if your cycle is very irregular’. The ad described ‘Menorota’ as ‘a MUST for all married women who wish to join the many thousands now LIVING NATURALLY, free from tension and anxiety for the first time’.^66^

The emphasis here on married women in the advertisements is revealing and illustrates the wider lack of approval for single women using family planning methods. Moreover, as Eimer Philbin Bowman’s 1977 study of first-time visitors to a Dublin family planning clinic showed, some Irish doctors were unhappy about prescribing the pill to unmarried women.^67^ Similarly, as Elizabeth Siegel Watkins has found in her study of the contraceptive pill in the United States, by the mid-1960s, many people frowned upon single women using any form of birth control because it implied that these women were not only having sex outside of marriage but also planning ahead for it.^68^

In a feature article by Mary Leland in the magazine in 1966 entitled ‘Whither love?’, which explored attitudes towards sex and love among married and single Irish women, one woman, Mrs. W.K., explained her regrets at her lack of knowledge about birth control when she got married. ‘I was terribly disappointed when I became pregnant three months after our marriage. I just wasn’t ready for it and I believe it must have been the grace of matrimony that helped me eventually to become happy about it’.^69^ The situation experienced by the women interviewed by Leland, in terms of access to contraception, produced an angry reaction, according to Leland, as they were restricted by both the medical profession and the priesthood:

Talking to these people, married and single, I sensed a kind of rage, mostly against the doctors or the priests, both of whom, it was felt, had confused the issues of sex, contraception and marriage so inextricably that it was difficult to decide on the truth. For the most part they shopped around, looking either for a sympathetic and realistic priest or a responsive and understanding doctor – often for both. ^70^

Leland’s comments here are indicative of the lived reality for many Irish women in the period. In order to gain access to the contraceptive pill, women first needed to find a sympathetic doctor. Moreover, if they were Catholic, the choice to use birth control meant either reconciling this with their own consciences or finding an understanding priest who would forgive them in confession. Similarly, in a subsequent article, Monica McEnroy drew attention to the idea of sympathetic doctors, stating, ‘a hopeful percentage of doctors are prescribing anovulants. They could help desperate women in the less enlightened areas’.^71^ McEnroy advised readers to contact her with the names of sympathetic doctors so that she could devise a list of doctors for ‘the women who send the sad fan mail to this page’. Readers were encouraged to ask their doctor if they would ‘help *Woman’s Way* to help readers whose family life or health is suffering from lack of reliable conception control’, with the understanding that their name would never appear in print and that the physicians should treat the women as ordinary patients.^72^

As for finding sympathetic priests, women’s experiences appear to have varied considerably. In 1973, the magazine described Maura’s situation. Maura, who had not told her husband she was taking the pill, went to her priest for advice. Somewhat surprisingly, he was sympathetic and although he advised that contraception was against Church teachings, he explained that ‘he personally believed that it was a matter for her own conscience’. Angela, a mother of four, went to her priest for advice and had a markedly different experience: ‘He was furious. He gave me a lecture about the evilness of contraception and how I would be flaunting the authority of the Holy Father’.^73^

The issue of sympathetic authority figures with regard to birth control continued to be a prominent theme of discussion in the magazine throughout the 1960s, in particular as Catholics waited for a definite response from Rome on the issue. In September 1966, the magazine printed the first article of a three-part series on the contraceptive pill and the moral implications of its use for Catholics. The first article, written by Rev. Denis O’Callaghan, acknowledged that the pill had many medical uses, such as for menstrual disorders or to ‘postpone a period that may occur at an inconvenient or embarrassing time’. In these situations, the author acknowledged that ‘there is no moral problem’ because the question of contraception did not arise. However, ‘the fact that the pill has these lawful uses increases the confusion’. O’Callaghan acknowledged that there were special cases where a doctor could lawfully protect a woman by prescribing her the pill, such as ‘where the girl in question is irresponsible and incapable of free consent’, or in the case of where a pregnancy would pose a severe threat to the life of the mother. In these cases, there was a clear principle for the prescription of the pill. Ultimately, however, he argued that ‘this can hardly be said of a number of other forms of special pleading and rationalizing which sympathetic doctors use to justify their employment of the Pill’.^74^

An unnamed Dublin gynecologist wrote the second article on the medical uses of the contraceptive pill. The article provided comprehensive details on how the pill worked for the lay reader, as well as outlining its potential side effects. While the side effects of the pill had been an important concern of second-wave feminists, the author played these down, noting that ‘these are infrequent and seldom persist after the first three courses’.^75^ Concluding, the gynecologist explained ‘the pill is a simple, reliable and aesthetic method of birth control. Side effects are infrequent and long-term risks unlikely. Medical supervision is necessary’.^76^ This gynecologist’s views again reinforce the perceived authority of the medical profession over women’s reproductive choices.

The final article, by Monica McEnroy, assessed the personal impact of the dilemma surrounding the contraceptive pill on Irish mothers.^77^ McEnroy wrote that she regarded both the lack of availability of contraceptive methods and doctors’ emphasis on encouraging patients to use the rhythm method to be a ‘national scandal’. In particular, she drew attention to the impact of the dearth of choice of contraceptive methods on women’s physical and mental health.^78^ In McEnroy’s view, it was up to Irish women to ‘stop being afraid’ and she encouraged women to speak out against the law and Church teachings on contraception:

We must stop being afraid of our husbands; afraid of our bishops; afraid of our doctors; afraid of everyone. If a little moral courage, backed by an honest conscience, could be drummed up in the young women in this country, we might bring this poor lopsided country of ours back to some sort of Christian common sense.^79^

Again, McEnroy highlighted the difficulties facing Irish women, and she noted that doctors, husbands, and Church representatives were the key figures standing in the way of women’s reproductive rights and choices. Although McEnroy was not calling for Irish women to reject their religion, she felt that it was the task of young women to stand up to these patriarchal structures. The magazine published a series of letters in response to the series in a later issue. Mrs. Rosemary R. Lucas, wrote ‘How can men presume to decide what is right for us?’, and praised the magazine for publishing the series on the pill. ‘Prospective wife’ also praised McEnroy’s ‘excellent article’, stating that she agreed ‘that is entirely between the couple concerned and God whether to use contraceptives or not’.^80^

Not all readers were sympathetic. Elizabeth Dalton, a mother of five children who declared that she had ‘been practicing the safe period since the first was born’, felt that the magazine had gone too far in publishing McEnroy’s piece on the pill. She stated ‘In a magazine like yours which is read by all types of women in all walks of life and consequently must have a large influence, such strong personal views on such an important issue must be handled with the greatest care’. This seems to suggest that Dalton was concerned about the issue of single women accessing information on contraception, and perhaps, the issue of young people reading about it, considering the wide readership of the magazine. Similarly, another reader, A.M., stated

Many of us Irishwomen still have faith and trust in God and loyalty to the Holy Father whose decision we shall accept as being God’s will for us. Monica McEnroy seems to be taking it upon herself to speak for Irishwomen. Well, she’s not speaking for me and I object to her censorious and ill-timed pontificating.^81^

Finally, while access and use of contraception was heavily influenced by patriarchal structures of authority, it also heavily depended on the dynamics of a marriage. *Woman’s Way* drew attention to cases where husbands interfered in women’s access to the contraceptive pill. One mother of four, writing to the advice column in 1968 explained that her doctor had stopped prescribing her the pill because her husband had ‘called in to object … on the grounds that “you have to take what is before you in life”’. The agony aunt stated ‘I think that both your doctor and your husband have forgotten that you are the person to decide. I suggest that you make this point quite firmly and cheerfully’.^82^ Some women believed that the success of the safe period was down to having a supportive husband. Speaking in 1968, Ethna Viney argued that ‘the rhythm method is unworkable in a marriage where communication between husband and wife is not good’.^83^ Similarly, according to Denise O’Rourke writing to the magazine in 1971, ‘if the husband is selfish about this aspect of marriage it often follows that he is mean and inconsiderate in other aspects too’.^84^ Moreover, two women interviewed for *Woman’s Way* magazine in 1973 explained that they had not told their husbands they were taking the contraceptive pill for fear of an argument or creating tension.^85^ Ultimately, a woman’s access to the contraceptive pill therefore largely depended, not only on class and location, but also on a sympathetic doctor, an understanding priest or a willingness to ignore Church teachings, as well as a supportive husband; essentially patriarchal goodwill. Therefore, it is unsurprising that so many women struggled to gain access to it.

## *Humanae Vitae* and religious conscience

As women’s interactions with priests suggests, the issue of religiosity and decisions around contraception were significant to Irish men and women, and throughout the early 1960s Irish Catholics awaited a decision from Rome regarding the Church’s stance on contraception. Indicative of such views are the attitudes of one twenty-four-year-old woman expecting her third child, who reporter Dorine Rohan interviewed in 1967. Rohan asked the woman if she would use the pill or any form of contraceptive if the Church allowed it, or whether she would use it without the Church’s permission. The woman explained, ‘Well, we’d rather have the permission. I wouldn’t like to have to give up my religion’.^86^ This suggests, for this particular woman, that she would rather have the Church’s permission, but there is an implication here that she would use it anyway and reconcile her choice with her moral conscience. Such debates surrounding the moral question of taking the contraceptive pill intensified following the publication of the Catholic Church’s encyclical *Humanae Vitae* in 1968, when the magazine began to discuss the issue of personal conscience in more detail. *Humanae Vitae*, the papal encyclical that banned artificial contraception, came at a crucial moment in terms of the history of birth control. The question of contraception had become the topic of heated debate within the medical profession and in the public arena, particularly with the advent of the contraceptive pill from the early 1960s. Many Catholics hoped that the Pope’s encyclical would constitute a more relaxed approach to the issue of birth control. However, *Humanae Vitae* reinforced the Church’s conservative views relating to the purpose of marriage and condemned all methods of artificial contraception.^87^ As Deirdre Foley has argued, ‘*Humanae Vitae* created a temporary obstacle for many Irish Catholics, particularly the less well-off, in accessing artificial methods of contraception (primarily the pill) in Ireland’.^88^ State law enforced Church teaching in the Republic of Ireland, and, as Peter Murray has noted, ‘it was around the law rather than the teaching that debate revolved once the initial impact of *Humanae Vitae* has been absorbed’.^89^

Following the introduction of the encyclical, Irish doctors now faced a new dilemma. According to one article in the *Irish Times* in 1968, ‘for a number of years now, since the introduction of the Pill, the doctors have been unwillingly carrying a certain moral responsibility’ in deciding whether or not to prescribe the pill to a patient.^90^ However, now that the moral position was made clear and Catholic couples were forbidden to use the pill, the article asked ‘has the responsibility of the Catholic doctor changed?’^91^

Following the introduction of the papal encyclical, *Woman’s Way* published a series of letters received on the subject of ‘The Pope and the Pill’. These letters provide insight into attitudes towards the encyclical among Irish women readers. Mrs. R.H. from Cork, who was almost thirty, explained that she had married at nineteen and had six children in eight years. She detailed the effects of the lack of access to contraception. She had been told by her physician ‘that I am almost certain to keep having children for the best part of the next twenty years if I am not careful’. R.H. asked ‘is it any wonder that there are so many women suffering from mental breakdown due to the strain of trying to manage on their incomes and living from month to month?’ Mrs. M.B.D. from Tullamore discussed the implications of the encyclical on ordinary Irish mothers. She stated that the safe period method caused ‘stress and strain that marriage in this day and age is unable to withstand’ and argued that ‘we all have our lives to live and the right to exercise our free will’. Mrs. Maura Hann from Arklow, who interestingly unlike most of the other women featured in the letters page, signed her letter with her full name, stated that the encyclical ‘was a bitter disappointment to the majority of Catholic couples’ with lack of access to contraception being ‘the chief cause of the unhappiness and apathy so prevalent in Irish homes to-day’. Others were more positive about the Pope’s proclamation and perhaps, as a consequence of their religious views, agreed with the encyclical. ‘Widow and mother of five sons’ stated that the whole world should thank Pope Paul ‘for the wonderful job he has made of the white paper on birth control’. Mrs. F. O’Sullivan asked ‘what is all the fuss about?’, stating that Catholics had never been allowed contraceptives and the Church should not be expected to engage in ‘bending over backwards to keep them Catholic by every means in her power that those who want to have their cake and eat it feel that the Pope should give his blessing on their promiscuity’.^92^

According to Monica McEnroy, the magazine received letters every week ‘asking where a doctor can be found who will prescribe the Pill’.^93^ In a piece outlining the debates around the world on the encyclical she pointed out that ‘Obeying *Humanae Vitae* will be little trouble to anybody in Ireland except the women who are being asked to take the health risks’.^94^ The magazine often discussed these health risks, featuring ‘hard cases’ of women who were struggling after multiple pregnancies and emphasized the theme of responsible parenthood. For instance, in 1971, Kate Kennelly’s article on family planning focused on cases of women who had had several pregnancies. Kennelly quoted an account by Dr. Eamon O’Dwyer, professor of obstetrics at University College Galway:

Mrs. X came to me when she had already had ten children and four abortions. She had no knowledge of the safe period and no interest in knowing about it because her husband insisted on his conjugal rights at all times. He was also a hopeless alcoholic, so she had to support the entire family on her own meagre earnings. I had no hesitation in prescribing the Pill for this woman, because she was a special case. Desperate though she was, she would not have had the knowledge to ask for it herself.^95^

Perhaps, for O’Dwyer, the prescription of the pill was the lesser of two evils if it would prevent the woman having further abortions. A letter to the magazine in March 1971 from Mrs. M. McD in County Cavan stated that while she was happy to be expecting her fourth child in five years, she knew ‘a woman who is expecting her seventh child. Her eldest is nine and she has had two miscarriages and a stillbirth as well. She has been attending a nerve specialist for four years’. Mrs. McD asked ‘Don’t you think that she is entitled to some form of contraception? I do’.^96^

The Catholic hierarchy in Ireland continued to emphasize the Church’s line on contraception in the wake of continuing debates on the legalization of contraception. Archbishop of Dublin, John Charles McQuaid had a pastoral read at Sunday mass in the Dublin diocese on 28 March 1971. This was in response to increasing public discussion of the issue of contraception in Ireland and the activities of legislators, such as Irish Senator Mary Robinson, who were attempting to liberalize the law. The pastoral, which was also published in Irish newspapers, declared that if legislation was passed allowing contraception, it would be ‘an insult to our Faith; it would, without question, prove to be gravely damaging to morality, private and public; it would be and would remain a curse upon our country’.^97^ McQuaid argued against the idea of contraception being a right, stating that ‘any such contraceptive act is always wrong in itself’, and suggested that the issue of making them available was one of ‘public morality’. Moreover, he argued that ‘the public consequences of immorality that must follow for our whole society are only too clearly seen in other countries’.^98^

Mrs. T.D. from Kilkenny wrote to the magazine to state that she was ‘very angry after having read Dr. McQuaid’s letter criticizing contraception’, stating that it was unfair that certain older rules of the Church such as fasting had been relaxed, while others had not. Similarly, Mrs. L. Morris from Fermanagh, expressed her disappointment in the clergy and their ‘little faith in Irish mothers’ and drew attention to the double code of morality in society that was ‘to blame for a lot of promiscuity’.^99^

Others were more supportive. Mrs. M.L. from Laois, the mother of ten children, stated that she fully supported the Church’s teaching on contraception and divorce and believed that the archbishop had ‘every right to speak out strongly on such objective moral laws’. According to Mrs. M.L.,

having successfully reared my children in difficult and often poor circumstances I find it difficult to understand young couples nowadays and their comparatively easy way of living, their attitude towards their Christian way of life as regards marriage, marital rights and so on.^100^

Arguments against the legalization of contraception often centered on fears around young people and promiscuity, suggesting that promiscuity was the next step along a slippery slope. Mrs. Geraldine Lynch from Dundalk stated that if contraceptives became legal, it would be ‘an open invitation for young couples who have become tired of drink, smoking and everything else?’.^100^ Similarly, Mrs. Marie C. Dunne wrote to the magazine asking ‘if contraceptives were to be sold over the counter legally what would happen to our unmarried youth who took advantage of it? It could be injurious to their health, apart altogether from the moral aspect’, adding that ‘a permissive society is a sick society and what sane person wants a sick society?’^101^ However, others such as Mrs. C.W. from Kildare believed that couples were entitled to receive a ‘sympathetic and understanding hearing from their doctor’ if they sought out advice on contraception, and encouraged the establishment of more family planning clinics so that privacy for the couple could be ensured.^102^

## Conclusion

In 1973, Irish magazine *Woman’s Way* published the testimony of several Irish women who were taking the contraceptive pill. One such woman was twenty-eight-year-old Clare, married for six years with four children, and who had been taking the pill for almost a year, and had not told her husband in order to avoid rows and tension in their relationship, as she believed he would not approve. She had tried the safe period but found it to be ‘utterly useless … in fact, my last two children were born while we were using this method’. Clare expressed her relief now that she was taking the pill, stating ‘It’s wonderful to know that I won’t become pregnant. I was very ill and had a difficult time carrying all my children. I think that I’d have had a nervous breakdown if I’d found myself pregnant again’.^103^

Discussions of family planning in the Irish media in the 1960s and 1970s focused on the contraceptive pill because it was removed from the act of sexual intercourse and in this way could be more easily discussed. Magazines such as *Woman’s Way* escaped censorship, perhaps the censorship board deemed them to be innocuous. They provided many Irish women with important information on family planning and how to access contraception. These articles also give us insight into the experiences of women living under the ban on contraception because they provided a forum for women to discuss their concerns and voice their views. Unlike other European countries, where the press tended to focus on the side effects of contraception, the work of journalists like Monica McEnroy in *Woman’s Way* helped to highlight the plight of Irish women’s lived realities for a wide audience.

Women’s access to the contraceptive pill in Ireland largely depended on several key factors which were influenced by gendered dynamics and the power of patriarchal structures. These included reconciling the decision to take the contraceptive pill with their religious faith (or finding a sympathetic priest) and finding a general practitioner who would prescribe it. Marital dynamics were also important, as were class and geographical location. Ultimately, however, to focus on these factors suggests a lack of agency of Irish women. It is evident that some Irish women did find means of accessing the contraceptive pill, yet, yet this was largely dependent on the cooperation of doctors, priests, and husbands. Ultimately, though, the wider discussion of the contraceptive pill, and the lively debates in *Woman’s Way* around it, suggest that many Irish women were beginning to display more agency in relation to birth control, and following the publication of *Humanae Vitae*, many were beginning to question the power of the Church over their reproductive choices.

## Notes

1 Tom Myler, ‘Women Who Live in Fear’, *Woman’s Way*, week ending Friday, December 7, 1973, 44.

2 For detailed accounts of the history of the Criminal Law Amendment Act and the role of the Catholic Church and Irish government in debates over contraception, see: Ann Daly, ‘Its Effect on Public Morality is Vicious in the Extreme: Defining Birth Control as Obscene and Unethical, 1926–32’, in *She Said She Was in the Family Way: Pregnancy and Infancy in Modern Ireland*, ed. Elaine Farrell (London: Institute of Historical Research, 2012); Sandra McAvoy, ‘A Perpetual Nightmare. Women, Fertility Control and the Irish State: the 1935 Ban on Contraceptives’, in *Gender and Medicine in Ireland 1700–1950*, eds. Margaret Preston and Margaret Ó hÓgartaigh (Syracuse, NY: Syracuse University Press, 2012), Chrystel Hug, *The Politics of Sexual Morality in Ireland* (Basingstoke: Palgrave Macmillan, 1999), 79–82 and Lindsey Earner-Byrne, ‘Moral Prescription: the Irish Medical Profession, the Roman Catholic Church and the Prohibition of Birth Control in Twentieth-century Ireland’, in *Cultures of Care in Irish Medical History, 1750–1950*, eds. Catherine Cox and Maria Luddy (Basingstoke: Palgrave, 2010).

3 McAvoy, ‘A Perpetual Nightmare’, p. 196. For instance, in September 1967, the Censorship of Publications Board banned British doctor Dr. J. F. Robinson’s book *The Contraceptive Pill*. ‘Books banned by censor’, *Irish Times*, September 28, 1967, 8.

4 Mary E. Daly, *Sixties Ireland: Reshaping the Economy, State and Society, 1957–1973* (Cambridge University Press, 2016), 144.

5 Daly, *Sixties Ireland*, 144–5.

6 Ibid.

7 Eurostat Fertility statistics, 1960–2018. https://ec.europa.eu/eurostat/statistics-explained/index.php/Fertility_statistics (accessed August 28, 2020).

8 Mary E. Daly, *The Slow Failure: Population Decline and Independent Ireland, 1920–1973* (University of Wisconsin Press, 2003), 4.

9 For an overview of debates concerning the contraceptive pill, see Laura Kelly, The Contraceptive Pill in Ireland c.1964–79: Activism, Women and Patient-doctor Relationships’, *Medical History* 64, no. 2 (April 2020), 195–218.

10 ‘3 Irishwomen in 100 using “the pill”’, *Irish Times*, June 29, 1967, 7.

11 Mary Maher, ‘A Short History of the Pill in Ireland’, *Irish Times*, March 14, 1968, 8.

12 Ibid.

13 Earner-Byrne, ‘Moral Prescription**’**. See also Earner-Byrne’s groundbreaking study, *Mother and Child: Maternity and Child Welfare in Dublin, 1922–60* (Manchester University Press, 2007).

14 Emilie Cloatre and Máiréad Enright, ‘“On the Perimeter of the Lawful”: Enduring Illegality in the Irish Family Planning Movement, 1972–1985’, *Journal of Law and Society* 44, no. 4 (2017): 471–500. More attention is given to government and medical debates surrounding family planning than the role of activists groups. See: Diarmaid Ferriter, *Occasions of Sin: Sex and Society in Modern Ireland* (London: Profile Books, 2009). For further discussion of the debates surrounding the Health Family Planning Act see: Aidan Beatty, ‘Irish Modernity and the Politics of Contraception, 1979–1993’, *New Hibernia Review* 17, no. 3 (2013): 100–118 and Brian Girvin, ‘An Irish Solution to an Irish Problem: Catholicism, Contraception and Change, 1922–1979’, *Contemporary European History* 27 (March 2018): 1–22. On the role of the medical profession and the IFPA, see: Mary E. Daly, *Sixties Ireland: Reshaping the Economy, State and Society, 1957*–*1973* (Cambridge: Cambridge University Press, 2016), 148–150 and Deirdre Foley, ‘“Too Many Children?”: Family Planning and *Humanae Vitae* in Dublin, 1960–1972’, *Irish Economic and Social History* 46, no. 1 (2019): 142–60.

15 On women’s reproductive rights activism in Ireland, see: Laura Kelly, ‘Irishwomen United, The Contraception Action Programme and the Feminist Campaign for Free, Legal, Contraception in Ireland, *c.*1975–1981’, *Irish Historical Studies* 43, no. 164 (2019): 269–297; Emilie Cloatre and Máiréad Enright, ‘Transformative Illegality: How Condoms ‘Became Legal’ In Ireland, 1990–1993’, *Feminist Legal Studies* 26, no. 3 (2018): 261–284; Mary Muldowney, ‘Breaking the Silence: Pro-choice Activism in Ireland Since 1983’, in *Sexual Politics in Ireland*, eds. Jennifer Redmond, Sonja Tiernan, Sandra McAvoy and Sonja Tiernan (Dublin: Irish Academic Press, 2015), 127–53; Ann Rossiter, *Ireland’s Hidden Diaspora: The Abortion Trail and the Making of a London-Irish Underground, 1980–2000* (London: IASC Publishing, 2009); Sandra McAvoy, ‘From Anti-amendment Campaigns to Demanding Reproductive Justice: the Changing Landscape of Abortion Rights Activism in Ireland 1983–2008’, in*The Unborn Child, Article 40.3.3 and Abortion in Ireland: Twenty-Five Years of Protection?* ed. J. Scwheppe (Dublin: Liffey Press, 2008), 15–45; Linda Connolly, *The Irish Women’s Movement: From Revolution to Devolution* (Basingstoke: Palgrave Macmillan, 2002); Anne Speed, ‘The Struggle for Reproductive Rights: A Brief History in its Political Context’, in *The Abortion Papers*, ed. Ailbhe Smyth (Dublin: Attic Press, 1992), 85–98.

16 See: Ciara Meehan, ‘The Irish Abortion Debate’, in *Crossing Troubled Waters: Abortion in Ireland, Northern Ireland, and Prince Edward Island*, eds. Colleen MacQuarrie, Claire Pierson, Shannon Stettner, and Fiona Bloomer (Island Studies Press, 2017), 20–47.

17 Cara Delay, ‘Pills, Potions, and Purgatives: Women and Abortion Methods in Ireland, 1900–1950’, *Women’s History Review* 28, no. 3 (2019): 479–99; Cara Delay, ‘Kitchens and Kettles: Domestic Spaces, Ordinary Things, and Female Networks in Irish Abortion History, 1922–1949’, *Journal of Women’s History* 30, no. 4 (2018): 11–34; Leanne McCormick, ‘“No Sense of Wrongdoing”: Abortion in Belfast, 1917–1967’, *Journal of Social History* 49, no. 1 (2015): 125–48; Lindsey Earner-Byrne and Diane Urquhart, *The Irish Abortion Journey, 1920–2018* (Basingstoke: Palgrave Macmillan, 2019), 51–68.

18 The magazine was published every fortnight from April 1963 up until August 1966 after which it was published weekly. Caitriona Clear, *Women’s Voices in Ireland: Women’s Magazines in the 1950s and 1960s* (London: Bloomsbury, 2016), 46.

19 The magazine was initially edited by Sean O’Sullivan. Clear, *Women’s Voices in Ireland*, 46, 52, 65.

20 Clear, *Women’s Voices in Ireland*, 46–8.

21 Ibid.

22 Ciara Meehan, ‘Has He Called You Frigid Lately? How Women’s Magazines Handled Sex and the Irish “Guilt Complex” in the 1960s’, *History Ireland* 26, no. 4 (July/August, 2018). https://www.historyireland.com/volume-26/issue-4-july-august-2018/has-he-called-you-frigid-lately/ (accessed online August 28, 2020).

23 Clear, *Women’s Voices in Ireland*, 4.

24 Ibid., 48, 81.

25 Ibid., 81.

26 Tracey Loughran, ‘Conditions of Illusion: Agency, Feminism, and Cultural Representations of Infertility in Britain, c.1960–80’, in *The Palgrave Handbook of Infertility in History*, eds. Gayle Davis and Tracey Loughran (Basingstoke: Palgrave, 2017), 433.

27 Ibid., 436.

28 Teresa Ortiz-Gomez and Agata Ignaciuk, ‘“Pregnancy and Labour Cause more Deaths than Oral Contraceptives”: The Debate on the Pill in the Spanish Press in the 1960s and 1970s’, *Public Understanding of Science* 24, no. 6 (2015): 658–71, on 658. The Franco regime banned contraception from 1941 to 1978. However, the contraceptive pill was available to Spanish women from the early 1960s as a therapeutic treatment for gynecological conditions and thus, like in Ireland, users evaded the ban on birth control there (Ortiz-Gomez and Ignaciuk, 659).

29 Agata Ignaciuk, ‘Love in the Time of El Generalisimo: Debates about the Pill in Spain before and after Humanae Vitae’, in *The Schism of ’68*, ed. Harris, 229–50, on 244.

30 Ibid.

31 Daly, *Sixties Ireland*, 148.

32 Foley, ‘“Too Many Children?”’, 144.

33 Declan Meagher and Dermot MacDonald, ‘A Hospital Family Planning Service’*, Journal of the Irish Medical Association* (December 1967), 443–5.

34 Ibid.

35 Mary Maher, ‘A Short History of the Pill in Ireland’, *Irish Times*, March 14, 1968, 8.

36 Letter from Kieran O’Driscoll, master NMH, dated 30 July 1968 (Dublin Diocesan Archives: McQuaid Public Affairs: xx/9–10).

37 Monica McEnroy, ‘Family Planning’, *Woman’s Way*, week ending Friday March 1, 1968, 18.

38 Ibid., 19.

39 Monica McEnroy, ‘Family Planning no. 2: The Politics of the Pill’, *Woman’s Way,* week ending March 8, 1968, 12–13.

40 Monica McEnroy, ‘Family Planning and the Law’, *Woman’s Way*, week ending March 15, 1968, 40–1.

41 Monica McEnroy, ‘The Sex Life of the Irish’, *Woman’s Way*, week ending Friday, August 2, 1968, 34–5.

42 ‘Women First: Contraception: What Do You Think?’, *Irish Times*, December 22, 1970, 6.

43 McAvoy, ‘A Perpetual Nightmare’, 196. The Lambeth Conference is a decennial meeting of Anglican bishops convened by the Archbishop of Canterbury.

44 Betty Hilliard, ‘The Catholic Church and Married Women’s Sexuality: Habitus change in late 20th century Ireland’, *Irish Journal of Sociology* 12, no. 2 (2003): 28–49, on 35.

45 Hilliard, ‘The Catholic Church and Married Women’s Sexuality’, 35.

46 Ortiz-Gomez and Ignaciuk, ‘Pregnancy and Labour’, 662.

47 Ibid., 662 and 664.

48 Ibid., 667.

49 Agata Ignaciuk, ‘Paradox of the Pill: Oral Contraceptives in Spain and Poland (1960s–1970s)’, in *Children by Choice?*, eds. Ann-Katrin Gembries, Theresia Theuke, and Isabel Heinemann (De Gruyter, 2018), pp. 95–111, on 108.

50 Tiago Pires Marques, ‘The Politics of Catholic Medicine: “The Pill” and Humanae Vitae in Portugal’, in *The Schism of ’68*, ed. Harris, 161–86, on 180.

51 Paul Ryan, *Asking Angela Macnamara: An Intimate History of Irish Lives* (Dublin: Irish Academic Press, 2012), 204–5.

52 ‘Science and Control by Angela Macnamara’, *Woman’s Way*, fortnight ending Nov. 30, 1963, 55.

53 Ibid.

54 Jennifer Redmond, ‘“Sinful Singleness”? Exploring the Discourses on Irish Single Women’s Emigration to England, 1922–1948’, *Women's History Review* 17, no. 3, 455–76, on p. 470. For more on this, see Jennifer Redmond, *Moving Histories*: *Irish Women's Emigration to Britain from Independence to Republic* (Liverpool University Press, 2018), Chapters 3 and 4.

55 Carole Holohan, *Reframing Irish Youth in the Sixties* (Liverpool University Press, 2018), p. 129.

56 Clear, *Women’s Voices in Ireland*, 89.

57 ‘Can You Help Me?’, *Woman’s Way*, first fortnight February, 1966, 49.

58 Clear, *Women’s Voices in Ireland*, 89.

59 Ryan, *Asking Angela Macnamara*, 197.

60 ‘Angela Macnamara gives a Helping Hand’, *Woman’s Way*, week ending Friday November 10, 1967, 59.

61 ‘Regulation of Family’, *Woman’s Way,* March 1, 1965, 21.

62 Ibid.

63 Ferriter, *Occasions of Sin,* 362.

64 Advertisement: ‘Warning: Family Planning’, *Woman’s Way*, week ending Friday September 23, 1966, 53.

65 Advertisement: Ovula, *Woman’s Way*, week ending Friday October 7, 1966.

66 Advertisement: Menorota, *Woman’s Way*, week ending Friday November 11, 1966, 41.

67 Eimer Philbin Bowman, ‘Sexual and Contraceptive Attitudes and Behaviour of Single Attenders at a Dublin Family Planning Clinic’, *Journal of Biosocial Science* 9, no. 4 (1977): 429–45, on 435.

68 Elizabeth Siegel Watkins, *On the Pill: A Social History of Oral Contraceptives, 1950–1970* (Baltimore: Johns Hopkins University Press, 1998), 2.

69 Mary Leland, ‘Whither Love?’, *Woman’s Way,* first fortnight August 1966, 44–5.

70 Ibid.

71 McEnroy, ‘Family Planning and the Law’, 40–1.

72
*Ibid.*

73 Heather Parsons, ‘Undercover on the Pill’, *Woman’s Way*, week ending Friday June 1, 1973, 8.

74 Rev. Denis O’Callaghan, ‘The Contraceptive Pill in Ireland: The Moral Situation’, *Woman’s Way*, week ending Friday September 9, 1966, 24–5.

75 Kelly, ‘Irishwomen United’, 281.

76 ‘The Contraceptive Pill in Ireland: A Doctor’s Report’, *Woman’s Way*, week ending Friday September 16, 1966, 24–5.

77 Monica McEnroy, ‘The Contraceptive Pill in Ireland: Personal Involvement’, *Woman’s Way*, 23 September, 1966, 9.

78 Ibid.

79 Ibid.

80 'The Contraceptive Pill: What Our Readers Think’, *Woman’s Way,* week ending Friday October 14, 1966, 23

81 ‘The Contraceptive Pill: What Our Readers Think’, 23.

82 ‘Marriage Guidance’, *Woman’s Way,* July 5, 1968, 23.

83 ‘Lives of Drudgery and Lack of Love’, *Irish Times,* April 19, 1968, 7.

84 ‘Over to You … ’ *Woman’s Way*, week ending Friday, April 23, 1971, 6.

85 Parsons, ‘Undercover on the Pill’, 8.

86 ‘Marriage Irish Style’, *Irish Times*, November 24, 1967, 10.

87 For more on the history of *Humanae Vitae*, see: Alana Harris, ed., *The Schism of ’68: Catholicism, Contraception and Humanae Vitae in Europe, 1945–75* (Palgrave, 2018).

88 Foley, ‘Too Many Children’, 160.

89 Peter Murray, ‘The Best News Ireland Ever Got? Humanae Vitae’s Reception on the Pope’s Green Island’, in *The Schism of ’68: Catholicism, Contraception and Humanae Vitae in Europe, 1945–75*, ed. A. Harris (Palgrave, 2018), 275–301, on 292.

90 ‘Doctors Dilemma about Prescribing’, *Irish Times*, July 30, 1968, 7.

91
*Ibid.*

92 ‘The Pope and the Pill’, *Woman’s Way*, week ending Friday September 6, 1968, 2–3.

93 ‘The Papal Encyclical’, *Woman’s Way,* week ending Friday September 13, 1968, 44–45.

94 ‘Dare the Pope Be Questioned?’, *Woman’s Way*, 8 November, 1968, 6–7.

95 Kate Kennelly, ‘Dilemma: Report on Family Planning’, *Woman’s Way*, week ending January 22nd 1971, 25.

96 ‘Over to You … ’, *Woman’s Way*, week ending Friday March 19, 1971, 6.

97 ‘Alteration of Law Would Be “A Curse Upon Our Country”: Archbishop’s Pastoral’, *Irish Times*, March 29, 1971, 11.

98 Ibid., 11.

99 ‘Over to You … ’, *Woman’s Way,* week ending Friday, May 14, 1971, 6.

100 ‘Over to You … ’, *Woman’s Way,* week ending Friday, May 28, 1971, 6.

101 ‘Over to You … ’, *Woman’s Way,* week ending June 4, 1971, 6.

102 ‘Over to You … ’, *Woman’s Way,* week ending Friday June 11, 1971, 6.

103 Parsons, ‘Undercover on the Pill’, 8.

